# Cutis Verticis Gyrata: A Secondary Form of a Rare Skin Condition Caused by Growth Hormone Therapy

**DOI:** 10.5334/jbsr.4005

**Published:** 2025-07-03

**Authors:** Brend Foriers, Philippe Demaerel

**Affiliations:** 1 Department of Radiology, University Hospitals Leuven, KU Leuven, Belgium

**Keywords:** Cutis verticis gyrata, Scalp, Growth hormone therapy, MRI

## Abstract

*Teaching point:* To recognize cutis verticis gyrata on MRI and raise awareness of growth hormone therapy as a possible cause.

## Case

**Figure 1:**
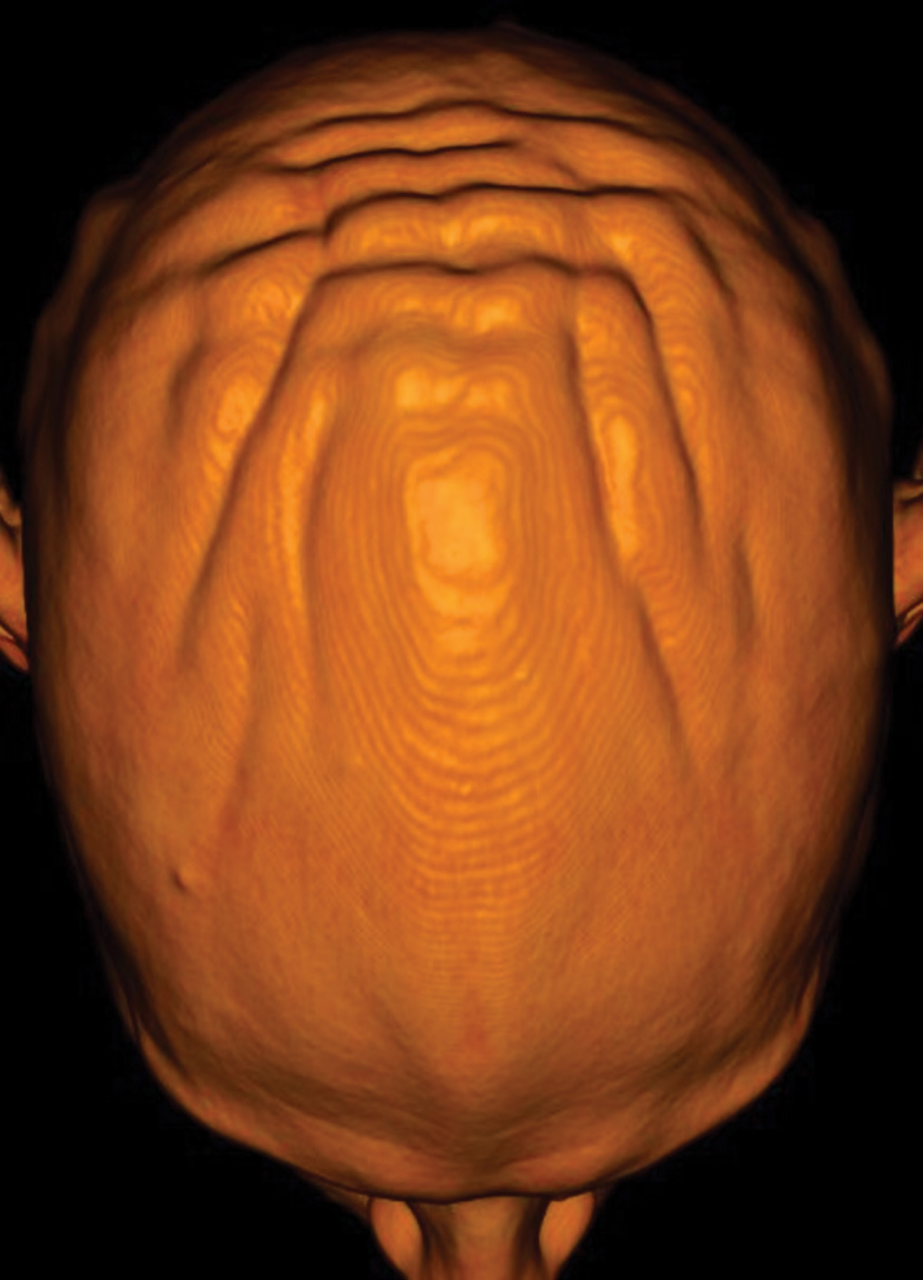
3D CT with cinematic rendering of the skin showing the anteroposterior orientation of the thickened skin folds.

**Figure 2:**
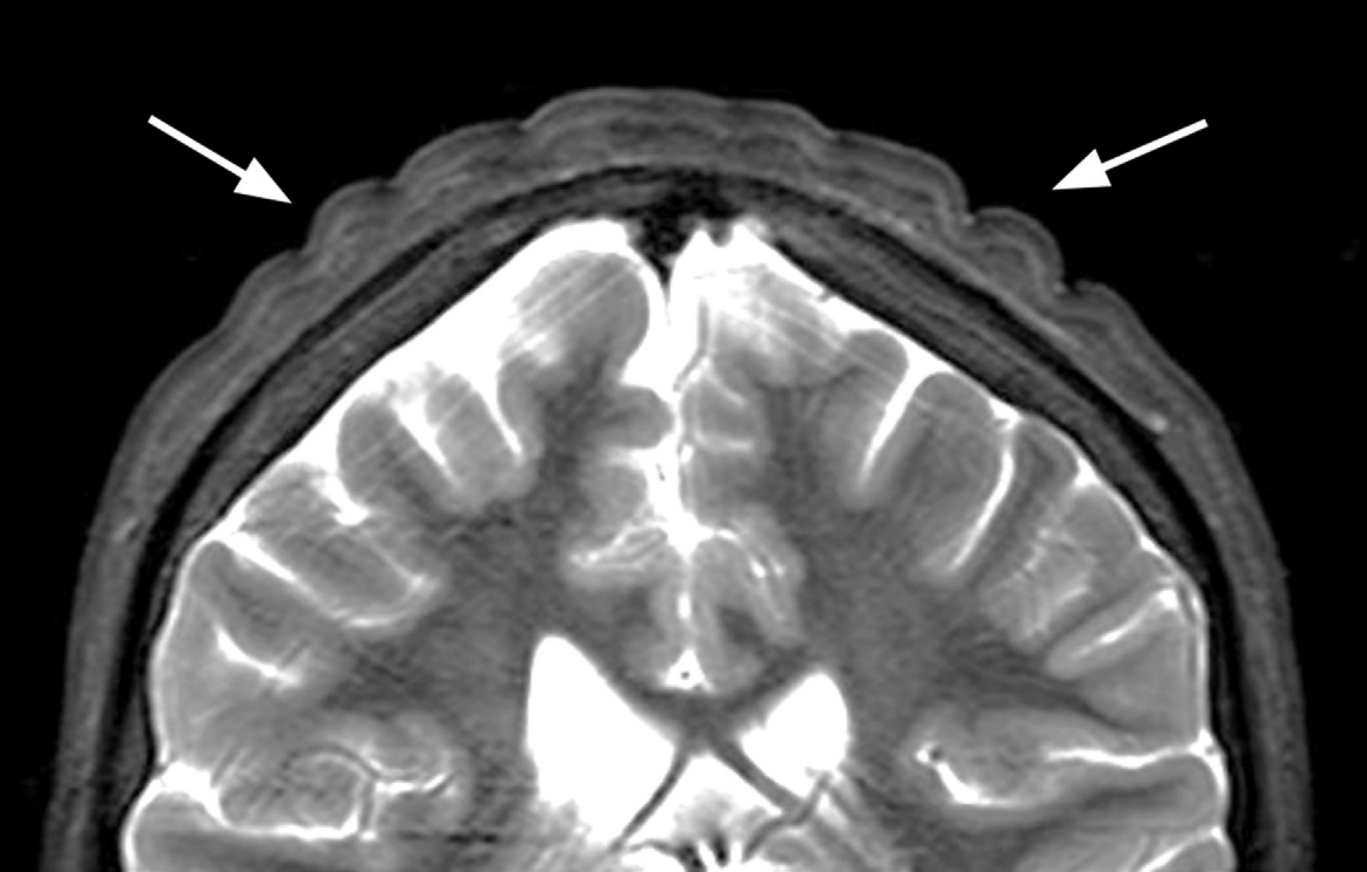
Coronal T2‑weighted image showing the thickened skin folds.

A 13‑year‑old boy with known Prader–Willi syndrome treated with growth hormone therapy (somatropin), since the age of 6 years, was presented to the outpatient pediatric endocrinology clinic with a groove‑shaped swelling of the scalp with anteroposterior orientation in the past 4 weeks (Figure 1). Brain MRI showed bilateral parietal groove‑shaped thickening of the cutis and subcutis in an anteroposterior orientation giving the scalp a ‘cerebral cortex like appearance’ (Figure 2). No intracranial abnormalities were seen. The imaging characteristics were fairly typical for cutis verticis gyrata (CVG).

**Figure 3:**
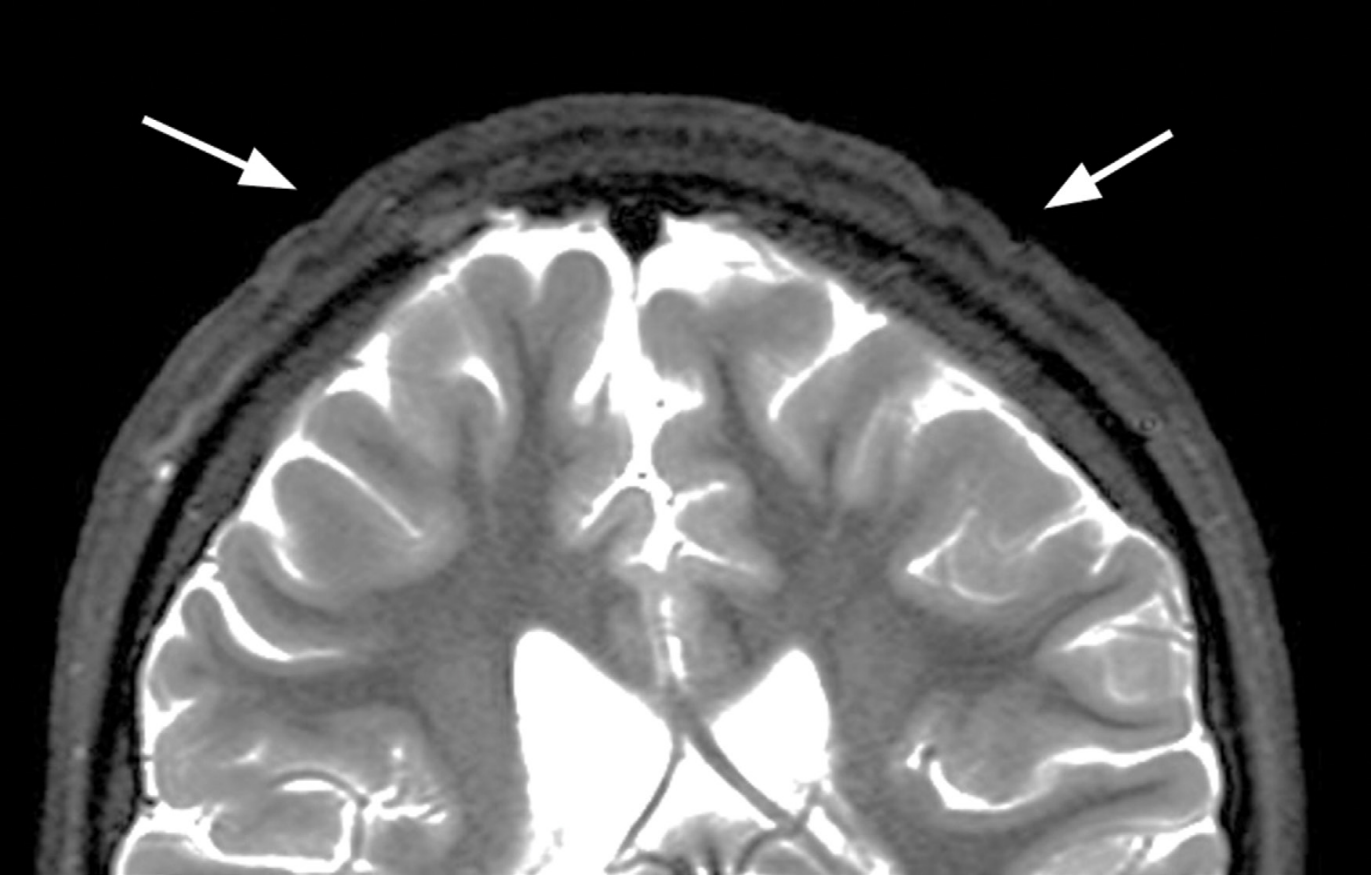
Coronal T2‑weighted image showing decreased thickening of skin folds 4 months after cessation of growth hormone treatment.

Growth hormone therapy was discontinued. Four months later, follow‑up brain MRI showed a decrease in the scalp abnormalities (Figure 3).

## Comment

Cutis verticis gyrata is a rare condition where the scalp develops abnormal folds, resembling the brain cortex. It is more common in males (1 in 100,000) than females (0.026 in 100,000), possibly because shorter haircuts make it easier to spot this abnormality in men [1].

High levels of growth hormone induce an excessive production of insulin‑like growth factor, which is the primary mediator of the proliferative effects. In the skin, this results in the overproduction of connective tissue and tissue edema.

CVG is categorized into two main forms: primary and secondary CVG. The primary form typically appears after puberty and tends to be symmetrical. Primary CVG is further divided into primary essential CVG, which is not linked to any other health issue, and primary non‑essential CVG, which is considered a neurocutaneous syndrome due to its possible associations with intellectual disabilities, epilepsy, seizures, and eye problems. In contrast to primary CVG, secondary CVG can occur at any age and appears with unevenly distributed folds. It is associated with various scalp conditions (such as eczema or moles) and systemic diseases (such as acromegaly or tuberous sclerosis), as well as certain syndromes such as Noonan and Klinefelter [1].

While CVG is primarily diagnosed clinically, it can also be discovered incidentally during CT and MRI scans, with characteristic thickening of the scalp skin and underlying tissues, due to an overgrowth of connective tissue and fat. The bony skull itself is unaffected [1].

The folds typically run from anterior to posterior. The longitudinal folds are best seen on coronal imaging, while sagittal imaging is more suited to observe any transverse folds. Mild cases, especially those only affecting the top of the head, might be missed if appropriate imaging views are missing. Although the diagnosis is generally straightforward, other conditions such as collagenoma or even tightly braided hair (‘corn rows’) can sometimes have similar appearances [1].

In our patient with Prader–Willi syndrome, there was a regression of the CVG following cessation of growth hormone therapy. Even though CVG is a benign and uncommon condition, understanding its unique appearance on skull imaging is crucial.
